# Urinary metabolomics using gas chromatography-mass spectrometry: potential biomarkers for autism spectrum disorder

**DOI:** 10.1186/s12883-022-02630-4

**Published:** 2022-03-17

**Authors:** Zaib Un Nisa Khan, Prem Chand, Hafsa Majid, Sibtain Ahmed, Aysha Habib Khan, Azeema Jamil, Saba Ejaz, Ambreen Wasim, Khaleel Ahmad Khan, Lena Jafri

**Affiliations:** 1 Department of Pathology and Laboratory Medicine AKU, Section of Chemical Pathology, Karachi, Pakistan; 2 Department of Pediatrics & Child Health AKU, Karachi, Pakistan; 3 Department of Pathology and Laboratory Medicine AKU, Karachi, Pakistan; 4 Bolan Medical College, Quetta, Pakistan

**Keywords:** Organic acids, Autism, Gas chromatography/mass spectrum

## Abstract

**Background:**

Diagnosis of autism spectrum disorder (ASD) is generally made phenotypically and the hunt for ASD-biomarkers continues. The purpose of this study was to compare urine organic acids profiles of ASD versus typically developing (TD) children to identify potential biomarkers for diagnosis and exploration of ASD etiology.

**Methods:**

This case control study was performed in the Department of Pathology and Laboratory Medicine in collaboration with the Department of Pediatrics and Child Health, Aga Khan University, Pakistan. Midstream urine was collected in the first half of the day time before noon from the children with ASD diagnosed by a pediatric neurologist based on DSM-5 criteria and TD healthy controls from August 2019 to June 2021. The urine organic acids were analyzed by Gas Chromatography-Mass Spectrometry. To identify potential biomarkers for ASD canonical linear discriminant analysis was carried out for the organic acids, quantified in comparison to an internal standard.

**Results:**

A total of 85 subjects were enrolled in the current study. The mean age of the ASD (*n* = 65) and TD groups (*n* = 20) was 4.5 ± 2.3 and 6.4 ± 2.2 years respectively with 72.3% males in the ASD group and 50% males in the TD group. Parental consanguinity was 47.7 and 30% in ASD and TD groups, respectively. The common clinical signs noted in children with ASD were developmental delay (70.8%), delayed language skills (66.2%), and inability to articulate sentences (56.9%). Discriminant analysis showed that 3-hydroxyisovalericc, homovanillic acid, adipic acid, suberic acid, and indole acetic were significantly different between ASD and TD groups. The biochemical classification results reveal that 88.2% of cases were classified correctly into ASD& TD groups based on the urine organic acid profiles.

**Conclusion:**

3-hydroxy isovaleric acid, homovanillic acid, adipic acid, suberic acid, and indole acetic were good discriminators between the two groups. The discovered potential biomarkers could be valuable for future research in children with ASD.

## Introduction

Autism spectrum disorder (ASD) is a complex neuro-developmental metabolic disorder involving multiple organ systems that can cause significant social, communication, and behavioral challenges in patients [1–3]. Many researchers report that there exist multiple factors involved in the pathogenesis of ASD including nutrients, infections, genetic disorders, and toxins [4–7]. ASD is highly genetically heterogeneous and both inheritable and de novo gene variations may contribute to its pathophysiology [8]. Since its discovery in 1943, ASD is still widely diagnosed through clinical and behavioral observation, due to a lack of reliable laboratory or radiological scans [1, 4, 9] In the past decade, numerous genes have been identified that contribute to the serious deficits in communication, social cognition, and behavior that individuals with ASD often experience [8]. ASD are categorized by impaired social interactions, difficulty in communications skills, repetitive behaviors, and additional stereotypical behavioral patterns [1]. Diagnosis is based on developmental history and examination of the patient using diagnostic criteria i.e. Diagnostic and Statistical Manual of Mental Disorders fifth edition (DSM-5 criteria) [10]. Behavioral tests like DSM-5 are subjective and time-consuming, require professional staff to be administered, and can only be used from age 3 years once the child is old enough to communicate. The purpose of identification of symptoms earlier is to provide earlier interventions that might improve outcomes [1].

Even though there are no objective means to diagnose ASD, it is significantly increasing in prevalence [11–13]. The publications on the prevalence of ASD have also increased over the last decade [13–16]. Several reasons may contribute to this rise, such as the definition of ASD has become broader, changes in the use of screening tools and diagnostic criteria, greater awareness, and recognition of ASD [15, 17]. Initially, in the 1970s, ASD was considered a rare disorder, and its prevalence was estimated to be in around 2 of 10,000 children [18]. In comparison to the USA, the prevalence of ASD was 99–116.1 per 10,000 in UK versus 34 per 10,000 in the USA [19]. The estimated prevalence of ASDs in Asia varied from 1.1 to 21.8 per 10,000 [17, 20–23]. According to the Pakistan Autism Society (PAS), in Pakistan, there are more than 350,000 children with ASD [24–26]. There are no national registries for ASD in Pakistan and there is a dearth of reliable data to know the exact incidence of ASD in Pakistan.

Organic acids are important metabolites in major metabolic pathways of fatty acids, carbohydrates, proteins, neurotransmitters, vitamins, co-factors, pathways involved in energy production (Krebs cycle), oxidative damage, and intestinal dysbiosis [27]. Few studies have demonstrated the utility of urine organic acid in children with ASD [28, 29]. Recent researchers have reported that several known neurometabolic disorders, inherited metabolic disorders (IMDs) and chromosomal alterations are thought to contribute to the development of ASD. Some of these metabolic disorders include phenylketonuria, disorders of purine metabolism, biotinidase deficiency, disorders of neurotransmitters, methylmalonic acidemias, and dicarboxylic aciduria [28–30]. There is also increasing awareness that oxidative stress may be implicated in the pathophysiology of ASD with evidence of oxidative stress markers in urine [31]. A study by Emond et al. showed that levels of glycolate, citrate and succinate were increased in the urine sample of ASD children [32]. Another study showed significant differences of 2-oxoglutaric, isocitric, citric, 4-hydroxybenzoic, adipic, 4-hydroxyphenyl acetic, hippuric, and suberic metabolites between ASD children and the control group [33]. There is no national newborn screening program in Pakistan for screening of IMDs.

There is not sufficient literature on the metabolomic data from urine organic acid testing in ASD and none from this part of the world [28, 34, 35]. The main aim of this study was to identify the metabolites and organic acids in the urine of children with and without ASD using gas chromatography-mass spectrometry (GC-MS).

## Material and methods

This case-control study was conducted in the Biochemical Genetics Laboratory (BGL) of Section of Chemical Pathology, Department of Pathology and Laboratory Medicine in collaboration with the Department of Pediatrics and Child Health, Aga Khan University Hospital (AKUH), Karachi Pakistan. This exploratory study was carried out from August 2019 to June 2021 after the approval from AKU’s ethical review committee (Reference number 2019–1213-4357). Our study is fully compliant with the Declaration of Helsinki [36]. The study cases (ASD group) were recruited from the Child Neurology Clinic and Child Development Program at the Department of Pediatrics and Child Health AKUH after the diagnosis of ASD was confirmed by a pediatric neurologist using the DSM-5 criteria [37] and after taking informed consent from parents or guardians of the children. Clinical history was obtained on a structured questionnaire including demographics, age at diagnosis of ASD, behavioral history, developmental history, and clinical history. None of them had any history of fever, vomiting, hypotonia, poor feeding, jaundice, and failure to thrive in the last 1 month before sampling. Children with known metabolic diseases, any recent hospital admission (< 1 month), presence of certain factors that would interfere with the detection of urine organic acids (compromised kidney function, hepatic insufficiency, dietary intervention therapy), diagnosis of other neuropsychiatric disorders, and children with any known inherited metabolic disorders for which treatment was received, were excluded from the study.

Healthy or typically developing (TD) children were selected randomly as controls, from children of laboratory employees who volunteered to participate in the study, without any history of learning and psychiatric abnormalities. For the control group, the same exclusion criteria were applied. Both cases and controls were selected through a convenient sampling method.

Midstream urine was collected in the first half of the day time before noon in sterile tubes without preservatives. The samples were placed on dry ice or in a freezer as soon as possible to avoid bacterial growth and transported to the Biochemical Genetics Laboratory (BGL), AKUH. The urine samples were frozen and stored at − 20 °C in aliquots until analysis. At the Biochemical Genetics Laboratory (BGL), before analyses, all urine sample concentrations were normalized with urine creatinine as a way of minimizing variability due to variability in urine concentration. Urine creatinine analysis (mmol/l) was performed for all samples on Siemens ADVIA 1800 by Jaffe’s kinetic method. One milliliter (ml) of undiluted urine was used for analysis in case of urine creatinine concentration < 1 mmol/l, while for samples with urine creatinine > 1 mmol/l, the volume of urine (patient/control) required for analysis was calculated by using eq. (1/ urine creatinine concentration of urine and 0.2 ml of deionized water was added to make up sample volume to 1 ml. One ml urine sample was taken in screw-capped glass tubes with a patient identification number. One hundred microliter of internal standard (3,3 dimethyl glutaric acid) was added followed by vortex and incubated at 60 °C in the water bath for 30 min and cooled to room temperature. The sample was then saturated with sodium chloride (NaCl) for oximation and mixed with a vortex mixer. Sample extraction was carried out with 50 μl of 6 M hydrochloric acid (HCL) and 5 ml of ethyl acetate. With help of the Pasteur pipette, upper ethyl acetate layered was transferred to a clean glass tube to which 10 μl of 25% ammonia was added. Vortexed briefly and dried down under nitrogen (oxygen-free) at room temperature for approximately 1 hour. For derivatization 80 μl of N, O-Bis-trimethylsilyl) trifluoroacetamide with 1% trimethyl chlorosilane and 20 μl of pyridine was added to dried extracts. Vortexed briefly and incubated at 75 °C for 30 min and cooled the tubes at room temperature. After cooling, 900 μl of iso-octane was added and mixed. The mixture was transferred to the micro-vial labeled with the patient’s identification numbers and screwed. Micro-vial containing the pre-treated sample was loaded into the auto sampler’s tray of GC-MS by Agilent Technologies United States. The Agilent Technology’s J&W Ultra Inert HP-5MS ultra inert capillary column was used with helium as carrier gas. Scan mode was created in the mass spectrometer acquisition software. The data generated from the mass detector was analyzed by Chem station software with the help of the following libraries: ORGASID, ACID97, and NIST. Semi quantitation of organic acids was done using the formula: area under curve of metabolite/area under curve of internal standard × concentration of internal standard. Organic acids were reported in pseudo units. Organic acid chromatograms of all study subjects were critically reviewed by credentialed chemical pathologists. During the period of study, the BGL successfully participated in multiple proficiency testing surveys from European Research Network for Evaluation and Improvement of Screening, Diagnosis, and Treatment of Inherited Disorders of Metabolism (ERNDRIM) and College of American Pathologists (CAP) for urine organic acids.

Statistical analysis was done using SPSS software (IBM SPSS, Statistics for Windows, USA) version 21.0. Mean and standard deviation were computed for quantitative data and frequencies were generated for qualitative data. The analytes for which we did not have pure standards were assigned a relative response factor that corresponded to the response factor for the internal standard. The height of each organic acid peak was interpreted as small, medium, and large with reference to internal standard peak height. The qualitative variables were compared with Chi-square statistics. To identify potential urinary biomarkers for ASD canonical linear discriminant analysis was carried out for the following organic acids that were quantified in comparison to internal standard: 3-hydroxyisovaleric acid, homovanillic acid, 2-ketoglutaric acid, 3- hydroxybutyric acid, adipic acid, suberic acid, 4-hydroxyphenyl acetic acid, indole acetic acid, lactic acid, and citric acid. This was an exploratory study and the level of statistical significance was defined as *p-*value < 0.05.

## Results

**Fig. 1:**
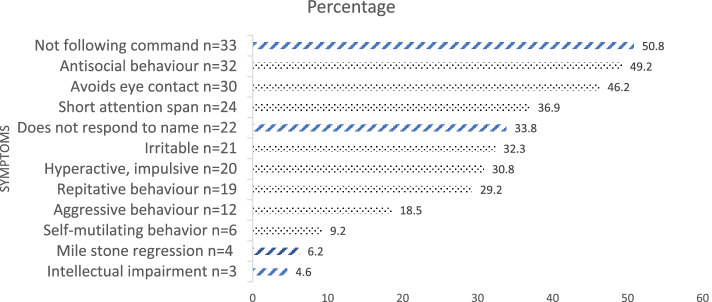
Bar chart showing behavioral and developmental symptoms observed in children (*n* = 65) with autism spectrum disorder (ASD). Diagonal bars represent developmental history; Dotted bars represent behavioral history

A total of 85 subjects were enrolled in the current study. The mean age of the ASD (*n* = 65) and TD (*n* = 20) groups were 4.5 ± 2.3 years and 6.4 ± 2.2 years, respectively. There were 72.3% (*n* = 47) were male subjects in the ASD group while there was 50% (*n* = 10) male in the TD group. Parental consanguinity was 47.7% (*n* = 31) and 30% (*n* = 6) in ASD and TD groups, respectively. In ASD 73.8% (*n* = 48), 9.2% (*n* = 6) and 16.9% (*n* = 11) were fed with formula, breastfeeding and mixed feeding in infancy, respectively. In TD 90% (*n* = 18) and 10% (*n* = 2) were fed with formula and mixed feeding in infancy, respectively. In the ASD group mean age at the time of diagnosis of ASD was 2.3 ± 1.5 years and 21.5% (*n* = 14) suffered from epilepsy. In ASD the common clinical signs noted were developmental delay (70.8%), followed by delayed language skills (66.2%) and inability to articulate sentences (56.9%), shown in Fig. 1. Physical examination, to rule out any gross morphological abnormality, conducted in both groups was unremarkable.

The mean urinary creatinine for ASD and TD groups were 5.2 ± 3.3 mmol/L and 6.2 ± 2.7 mmol/L, respectively. The urinary creatinine correlated with age in ASD (*r* = 0.31, *p* value 0.009) and not in TD group of children. Table 1 describes the organic acid peaks identified on chromatograms after analysis on GCMS. Comparison of organic acid peaks in ASD and TD groups separately using chi-square analysis showed a statistically significant difference in the following organic acids: aconitic acid, succinic acid, citric acid, indole acetic acid, palmitic acid, suberic acid, lactic acid, 2- ketoglutaric acid, adipic acid, hippuric acid, 5-hydroxymethyl-2-furoic acid, and pimelic acid. The urine organic acids that showed negative weak correlation with age were homovanillic acid (*r* = − 0.30, *p* value 0.004) and suberic acid (*r* = − 0.37; < 0.0001). The rest of the urine organic acids did not correlate with age. A sub-analysis was done to determine if gender differences affected any metabolite, no statistically significant effect of gender was seen on the urine organic acids.

**Table 1:** Distribution of urinary organic acid on GC-MS chromatograms amongst autism spectrum disorder (ASD) (*n* = 65) in comparison to typically developing (TD) children (*n* = 20) using chi-square analysis

Urine Organic Acid Peaks	Autism Spectrum Disorder (ASD) n (%)				Typical Developing (TD) n (%)				***p*** value
	Overall Peak	Small Peak	Medium Peak	Large Peak	Overall Peak	Small Peak	Medium Peak	Large Peak	
**Aconitic acid**	64(98.4)	55(84.6)	08(12.3)	01(1.5)	13(65)	11(55)	–	02(10)	< 0.0001
**Succinic acid**	64(98.5)	64(98.5)	–	–	02(10)	02(10)	–	–	< 0.0001
**Indole acetic acid**	59(90.8)	59(90.8)	–	–	5(25)	5(25)	–	–	< 0.0001
**Suberic acid**	56(86.2)	56(86.2)	–	–	01(5)	01(5)	–	–	< 0.0001
**Lactic Acid**	51(78.5)	51(78.5)	–	–	2(10)	2(10)	–	–	< 0.0001
**Adipic acid**	45(69.2)	45(69.2)	–	–	03(15)	03(15)	–	–	< 0.0001
**Hippuric acid**	43(66.2)	25(38.5)	8(12.3)	10(15.4)	02(10)	–	–	2(10)	< 0.0001
**Citric acid**	61(93.9)	25(38.5)	20(30.8)	16(24.6)	19(95)	17(85)	–	02(10)	0.001
**5-hydroxymethyl-2-furoic acid**^a^	24(36.9)	24(36.9)	–	–	0	–	–	–	0.001
**Palmitic acid**^a^	56(86.1)	53(81.5)	01(1.5)	02(3.1)	0	–	–	–	0.002
**Pimelic acid**^a^	21(32.3)	21(32.3)	–	–	0	–	–	–	0.002
**2- ketoglutaric acid**	47(72.3)	41(63.1)	05(7.7)	01(1.5)	20(100)	19(95)	–	1(05)	0.005
**3- hydroxybutyric acid**	54(83.1)	54(83.1)	–	–	19(95)	19(95)	–	–	0.181
**Oxalic acid**	14(21.5)	14(21.5)	–	–	03(15)	03(15)	–	–	0.523
**3-hydroxyisovaleric acid**	62(95.4)	62(95.4)	–	–	19(95)	19(95)	–	–	0.943
**Vanillylmandelic acid (VMA)**	62(95.4)	62(95.4)	–	–	20(100)	20(100)	–	–	1.000
**Homovanillic acid (HVA)**	65(100)	65(100)	–	–	20(100)	20(100)	–	–	1.000
**Phosphoric acid**	65(100)	64(98.5)	–	01(1.5)	20(100)	20(100)	–	–	1.000
**Tartaric acid** ^a^	1(1.5)	1(1.5)	–	–	0	–	–	–	1.000
**4-hydroxyphenyl acetic acid**	63(96.9)	47(72.3)	09(13.8)	07(10.8)	20(100)	14(70)	06(30)	–	1.88

Significant level at *p* < 0.05. ^a^Fisher Exact test

Mean comparison (pseudo units) was statistically significant for the following organic acids in ASD versus TD group: 3-hydroxyisovaleric acid (0.2 ± 0.14 vs. 1.05 ± 1.1), homovanillic acid (10.2 ± 6.7vs. 6.6 ± 2.4), adipic acid (1.54 ± 2.4 vs. 0.015 ± 0.04), suberic acid (1.3 ± 2.3 vs. 0.005 ± 0.02) and indole acetic (0.6 ± 0.9 vs. 0.25 ± 0.04), respectively and these organic acids were good discriminators between ASD and TD groups, as the separations were large. The 3-hydroxyisovaleric acid was low in quantity in ASD as compared to TD children while homovanillic acid, adipic acid, suberic acid, and indole acetic were higher in ASD than the TD group. This comparison is further described in Table 2. Table 2 also presents the standardized canonical discriminant function coefficients. The standardized canonical discriminant function coefficients show all quantitative variables in the model on the same scale and provide an index of the importance of each predictor (urine organic acids). 3-hydroxy Isovaleric score was the strong predictor followed by homovanillic acid, indole acetic, adipic acid, and suberic acid. These five variables (urine organic acids) with large coefficients stand out as those that strongly predict allocation to the ASD and TD groups. 2-ketoglutaric acid, 3-hydroxybutyric acid, adipic acid, suberic acid, 4-hydroxyphenyl acetic acid, and citric acid scores were less successful as predictors. Canonical correlation of 0.707 suggests the model explains 49.98% of the variation in the grouping variable, i.e., whether ASD or TD, while 50% remains unexplained. Wilks’ lambda indicates the significance of the discriminant function, it shows a highly significant function (*p* < .0001) and provides the proportion of total variability not explained, i.e., it is the converse of the squared canonical correlation (R2).

**Table 2:** Comparison between autism spectrum disorder (ASD) and typically developing (TD) children of the urinary metabolites using standardized canonical discriminant function

Urine organic acids in pseudo units	ASD	TD	Test of Equality of Group Means			Discriminant Function Coefficients
	Mean (SD)	Mean (SD)	Wilk’s Lambda	F	Sig.	
**3-hydroxy isovaleric**	0.20(0.14)	1.05(1.12)	0.697	36.035	< 0.0001	−0.812
**Indoleacetic acid**	0.61 (0.91)	0.02 (0.04)	0.908	8.455	0.005	−0.342
**Adipic acid**	1.54(2.38)	0.01 (0.04)	0.911	8.095	0.006	0.277
**Suberic acid**	1.28(2.35)	0.005 (0.022)	0.934	5.849	0.018	0.148
**Homovanillic acid**	10.07(6.8)	6.60(2.41)	0.941	5.178	0.025	0.404
**3-hydroxy butyric acid**	0.57(0.72)	0.25 (0.17)	0.955	3.889	0.052	0.107
**Lactic acid**	1.71 (1.37)	0.79 (3.46)	0.964	3.137	0.080	0.568
**Citric acid**	34.80(39.1)	17.58 (46.12)	0.968	2.725	0.103	0.052
**2-ketoglutaric acid**	5.98(12.04)	3.62(11.64)	0.993	0.598	0.442	−0.146
**4-hydroxyphenyl acetic acid**	32.40 (56.4)	23.57(33.2)	0.995	0.441	0.509	−0.015

The percent classification using discriminant function analysis results showed the sensitivity and specificity (95% CI) of urine organic acid to diagnose ASD were 100% (94.48 to 100) and 50% (27.2 to 72.8) respectively. According to the discriminant analysis 88.2% of cases were classified correctly into ‘ASD’ or ‘TD’ groups using urine organic acids (Table 3).

**Table 3:** Percent of correct classification of autism spectrum disorder (ASD) using discriminant function analysis

ASD_1			Predicted Group Membership		Total n(%)
			Yes n(%)	No n(%)	
**Original**	**Count**	**Yes**	65 (100)	0	65 (100)
		**No(TD)**	10 (50)	10 (50)	20 (100)

88.2% of original grouped cases correctly classified

## Discussion

In the omics era breakthrough advancements in the fields of genomics, proteomics, and metabolomics are adding to the data on ASD diagnostics. The profiling of small-molecule metabolites also known as metabolomics can provide means to identify disturbances in metabolic pathways in ASD [34, 38]. The metabolome reflects the interaction between genetic and environmental influences and can provide information to bridge the gap between genotype and phenotype in children suffering from ASD [28, 34, 35]. In this study the urinary creatinine correlated with age in ASD (*r* = 0.31, p value 0.009) which could be because of increasing muscle mass as age increases [39]. The analysis of the current study showed that children with ASD were more likely to have increased levels of homovanillic acid, indole acetic acid, adipic acid, and suberic acid. The modeling was performed using the discriminant analysis and canonical correlation for 10 urine organic acids to identify the potential biomarkers for ASD. The 3-hydroxy isovaleric acid (lower in ASD), homovanillic acid, adipic acid, suberic acid, and indole acetic were observed as good discriminators between the two groups. Our study findings showed that two dicarboxylic acids (adipic and suberic) were good discriminators between ASD and TD groups. The reason for the increase in these dicarboxylic acids in ASDs is unknown but from a biochemical point of view, the concentration of this acid might rise because of an increase in omega (ɷ) oxidation, which may be because of impaired β-oxidation of fatty acids in ASDs [40, 41]. Beta-oxidation occurs in the mitochondria and when mitochondrial metabolism is impaired, β-oxidation can also be disturbed. A Polish group of researchers observed the levels of succinic acid, adipic acid, and suberic acid in the urine of children with ASD before and after vitamin B supplementation and the results showed that after supplementation the levels were reduced in children with ASD [33]. Excessive intake of adipic acid-containing foods such as gelatin-containing desserts including fruit gels, jelly, puddings, and no-bake cream pies may also be a contributor which was not evaluated in our study. Suberic acid, a dicarboxylic acid, is present in the urine of individuals with fatty acid oxidation disorders. Elevated levels are found in individuals with medium-chain acyl-CoA dehydrogenase deficiency [42]. It is also associated with carnitine-acylcarnitine translocase deficiency, malonyl-Coa decarboxylase deficiency. Moreover, adipic acid is also found to be associated with 3-hydroxy-3-methylglutaryl-CoA lyase deficiency, carnitine-acylcarnitine translocase deficiency, malonyl-Coa decarboxylase deficiency, and medium Chain acyl-CoA dehydrogenase deficiency [43]. Adipic acid is also a microbial metabolite found in Escherichia [44]. However, to date, it has not been possible to ascertain whether increased adipic and suberic acids may be involved in the pathogenesis or symptomatology of ASDs in children. Whether dicarboxylic acids cross the blood-brain barrier and disturb the central nervous system in ASD children remains to be established [40].

Indole acetic acid was also elevated in our ASD group, as in a previous study using hydrophilic interaction chromatography (HILIC)-UHPLC and mass spectrometry approach [35]. It is an intermediate metabolite of tryptophan metabolism, which in turn is a precursor of serotonin. Bacterial species (*Clostridia species, Escherichia coli, Proteus Vulgaris, Paracolobactrum coliform, Achromobacter liquefaciens, and Bacteroides spp*) express tryptophanase which is responsible for transforming tryptophan into indole derivatives (indolyl 3-acetic acid and indolyl lactate) [38, 45]. There is increasing evidence of a brain-gut–microbe connection, and their regular cross-communications [45]. Many children with ASD generally suffer from gastrointestinal (GI) symptoms that can be comparable to those of irritable bowel syndrome but the exact prevalence of GI symptoms in autism is not known, with estimates ranging widely from 9 to 70% [45–47]. Studies have also shown a strong correlation between GI dysfunction and autism severity, across all domains including speech, social, and behavior [45, 47]. In one study, children with regressive autism were treated with a 6-week course of oral vancomycin, an antibiotic active against clostridia. Significant improvement in neurobehavioral and GI symptoms was noted in eight of the ten children studied providing evidence in favor of a toxin-producing Clostridium as a potential cause of regressive autism [47]. Daneberger et al. reported that children with ASD showed changes in urinary organic acid spectra which could be useful as biomarkers for indicating specific changes in the gut microbiota [48].

Our study also showed significant differences in the level of homovanillic acid (an intermediate metabolite of dopamine metabolism) between the ASD and TD groups, consistent with studies done by Federica Gevi et.al [34]. This finding suggests potential dopaminergic abnormalities in the investigated ASD group, which can explain disturbances of functioning of the dopaminergic system (repeated behaviors, mood disorders, aggression attacks, and disorders of social relationships) noted in children with ASD [1, 34, 49]. Urinary homovanillic acid correlated with impairment in neurobehavioral function assessed by the World Health Organization Neurochemical Core Test battery [50]. In support of this finding, studies have shown that vitamin B6 supplementation reduced the concentrations of urinary homovanillic acid and significantly reduced neurological symptoms including improvements in learning skills, reduction in hyperactivity and seizures [51–53].

The current study has some limitations. This was a single centered study and the number of enrolled ASD, and TD children was small, which is not helpful in creating a definitive model for ASD prediction using urine organic acid.. Second gastrointestinal disorders and dietary habits were not evaluated by standardized specific tools in ASD children. In this study following confounding factors such as dietary intake and environmental factors in both children with ASD and TD children were not controlled. Subgroup analysis could not be done for the severity of ASD, symptomatology, ethnicity, cognitive level, to explain the pathogenesis of ASD more extensively. There is a need to conduct similar metabolomic studies on larger sample size focusing on the correlation of metabolomic profiles and ASD phenotypic changes and severity.

## Conclusion

The early diagnosis and therapeutic intervention improve long-term outcomes in ASD. Our findings open new perspectives for a better understanding of the correlation between the clinical phenotype of children with ASD and their urine metabolome. Concentration (as evident by height and number of peaks) of aconitic acid, succinic acid, citric acid, indole acetic acid, palmitic acid, suberic acid, lactic acid, 2-ketoglutaric acid, adipic acid, hippuric acid, 5-hydroxymethyl-2-furoic acid, and pimelic acid was significantly higher in ASD as compared to TD children. However, the five organic acids that were good discriminators between ASD and TD groups were 3-hydroxy isovaleric acid, homovanillic acid, adipic acid, suberic acid, and indole acetic. Further larger metabolomic studies are required to study the etiology of ASD and to find the accurate diagnostic biomarker for this complex disorder.

## Data Availability

The datasets used and/or analyzed during the current study available from the corresponding author on reasonable request.

## Abbreviations

ASD: Autism spectrum disorders
TD: Typically developing
DSM-5 criteria: Diagnostic and Statistical Manual of Mental Disorders 5th edition
BGL: Biochemical Genetics Laboratory
CAP: College of American Pathologists
GC-MS: Gas Chromatography-Mass Spectrometry
ERNDRIM: European Research Network for Evaluation and Improvement of Screening, Diagnosis, and Treatment of Inherited Disorders of Metabolism
